# 174 A qualitative exploration of the influence of formal and informal care practices on physical activity in care home residents

**DOI:** 10.1093/eurpub/ckae114.036

**Published:** 2024-09-26

**Authors:** Gavin Wylie, Thilo Kroll, Miles Witham, Jacqui Morris

**Affiliations:** University Of Dundee, Dundee, United Kingdom; University College Dublin, Dublin, Ireland; Newcastle University, Newcastle, United Kingdom; University Of Dundee, Dundee, United Kingdom

## Abstract

**Background:**

Physical activity (PA) levels among older people who live in care homes are low, with residents estimated to spend 70 - 80% of their waking time inactive. Low PA in care homes reduces independence, function, and quality of life. Influences on PA behaviour stem partly from residents’ reduced intrinsic physiological capacity. However, influences on PA behaviour are also context-specific, and staff practices related to the organisational and cultural environments of care homes are critical in how (or if) care home residents engage in PA.

**Objective:**

To explore the role and perceptions of staff in the promotion of PA in care home residents.

**Methods:**

A focused ethnographic study in five care homes for older people comprised 54 hours of non-participant observation and 15 in-depth interviews with care home care workers. Observations of PA behaviours focused on key moments and the daily routines of the care homes. Interviews were informed by observations and sought explanations for observed behaviour. All data were analysed using thematic analysis informed by a social-ecological perspective.

**Results:**

The findings show how care workers perceptions of roles, identity, and sense of purpose regarding PA facilitation led to a continuum of formal and informal practices. Opportunities for PA appeared to be constrained or facilitated by care workers conceptualisations of the nature and purpose of care that stemmed from: (i) beliefs around how (or if) carers could facilitate PA opportunities within the constraints of the care routine; (ii) carers’ notions of identity and the purpose of their role; (iii) the boundaries of care work; (iv) formal practices in PA; (v) carers’ knowledge and understanding of PA.

**Conclusions:**

Care staff are the most appropriate people to sustain facilitation of PA on a regular basis, however, this is contingent on addressing detailed individual, interpersonal, organisational, and physical environmental influences on PA. The findings are placed in the context of a social-ecological model that can be used to support the development of interventions to increase PA in care homes. These findings provide evidence to support potential transformation of care worker roles to emphasise PA facilitation as a key part of the role.

